# A comparison of two different anthelmintic treatment regimens against natural gastrointestinal nematode infections on two Lithuanian sheep farms

**DOI:** 10.1186/s13028-017-0336-6

**Published:** 2017-10-11

**Authors:** Tomas Kupcinskas, Inga Stadaliene, Algimantas Paulauskas, Pavelas Trusevicius, Saulius Petkevicius, Johan Höglund, Mindaugas Sarkunas

**Affiliations:** 1 Department of Veterinary Pathobiology, Veterinary Academy, Lithuanian University of Health Sciences, Tilzės str. 18, 47181 Kaunas, Lithuania; 2 Faculty of Natural Sciences, Department of Biology, Vytautas Magnus University, 44404 Kaunas, Lithuania; 3 Buivydiskiai Veterinary Clinic, Kliniku str. 1, Buivydiskių k., 14165 Vilnius, Lithuania; 4 Department of Biomedical Sciences and Veterinary Public Health, Swedish University of Agricultural Sciences, Section for Parasitology, Box 7036, 750 07 Uppsala, Sweden

**Keywords:** Anthelmintic resistance, Nematode, Sheep, Targeted selective treatment, Targeted treatment

## Abstract

**Background:**

According to targeted treatment (TT), the whole flock is dewormed based on knowledge of the risk, or parameters that quantify the mean level of infection, whereas according to targeted selective treatment (TST), only individual animals within the grazing group are treated, based on parasitological, production and/or morbidity parameters. The aim of this study was to compare two different treatment protocols on sheep farms in Lithuania. The study was conducted from 15 April to 31 October 2014 on three sheep farms. On the TT (the whole flock) and T(S)T (with FECs ≥ 300, respectively) farms all adult animals were treated orally with fenbendazole irrespective of EPG counts before the grazing season. The second treatment was applied with injectable ivermectin on both farms. However, on the TT farm all sheep were also treated on 2nd August regardless of their EPG counts, while on the T(S)T farm only those animals with an EPG ≥ 300 were treated on 1 July using a threshold of ≥ 300 EPG. No treatments were administered on the control farm (n = 1) during the study.

**Results:**

Spring treatment of ewes significantly reduced nematode faecal egg counts (FEC) both on the TT and T(S)T farms, with the benefit of lowering pasture contamination with infective L_3_ stage larvae at the start of grazing season, while it remained significantly higher on the control farm. The positive effect of the spring treatment of ewes was reflected by increased body weight gains (BWG) in lambs in the first half of the grazing season. Following the second treatment, the weight gains in lambs on the T(S)T farm were higher compared to lambs on the TT farm, while BWG in the control lambs started to decrease. The difference was also substantiated by the body condition scores (BCS) and dag scores (DS) of lambs, which were highest on the T(S)T farm compared with those on the control and TT farms.

**Conclusions:**

The results of this study show that both treatment strategies were useful in reducing clinical effects (BCS and DS) of gastrointestinal nematode parasitism and increasing the performance in lambs. Furthermore, on the T(S)T farm some of animals were left *in refugia*, helping to slow down the development of anthelmintic resistance (AR) in future.

## Background

Gastrointestinal nematodes (GIN) have a worldwide distribution and are one of the main constraints in small ruminant production on grass [1–3]. Due to intensive use of anthelmintics, under-dosing and repeated treatments with the same anthelmintics, anthelmintic resistance (AR) presents an increasing challenge also in mainland Europe [4]. Anthelmintic resistance to each of the three established anthelmintic families has been recorded in GIN of sheep in many European countries, mainly against benzimidazoles [5–18], but recently also to macrocyclic lactones [7–11, 14, 16–18] and occasionally also to imidazothiazoles [8, 15, 16]. In United Kingdom there are also records of multiresistant worm populations [9, 10]. Reasons for applying TT and TST approaches is to effectively control nematode-induced production impacts, while preserving anthelmintic efficacy by maintaining a pool of untreated parasites *in refugia* [19]. More specifically TST approaches are applied to prolong the efficacy of anthelmintics and to avoid development of AR [20, 21]. Based on management strategies that employ *refugia*-based methods in which only a proportion of the flock is treated at any one time [21, 22]. According to TST the ability to effectively target anthelmintic use relies on the identification of those animals that will benefit most from treatment [22]. Accordingly TST requires diagnostic markers of GIN infection based on parasitological markers (such as faecal egg counts FEC or specific antibody levels), pathophysiological markers (such as diarrhoea and anaemia), production parameters (such as weight gain or milk and wool production), and/or morbidity parameters (such as BCS) [2, 21–25]. However, each specific TT/T(S)T strategy must be adjusted to local farming conditions. Important challenges are to define the combination of diagnostic marker(s) that can be used for identification of the groups or individuals that need to be treated, but also to determine treatment thresholds [26–28].

The aim of this study was to compare and to evaluate two different treatment systems on two sheep farms (a TT regimen *versus* a T(S)T) and to compare these with the situation on an untreated control farm using parasitological (FEC), production (BWG) and morbidity markers (BCS, DS) in growing lambs.

## Methods

### Trial design

The study was conducted during the grazing period from 15 April to 31 October 2014. Three sheep farms in the district of Prienai (54.72509, 24.049087 (WGS)) in the southern Lithuania were enrolled. On the TT and T(S)T farms, different treatment regimens were applied based on FEC, while no treatments were administered on the control farm during the course of the study. The T(S)T strategy consisted of: first a TT of the whole flock (before turn-out in spring) and then a TST but only of those animals with trichostrongylid FEC ≥ 300 eggs/g faeces (EPG) in early July. The size of the flocks was 24, 57 and 28 animals, respectively. The farms were comparable in terms of grazing density (6–10 animals per hectare, including lambs), sheep breeds (i.e. Lithuanian black-headed, Romanov and crossbreeds), location (the distance between the farms was max. 15 km) and a set stocked grazing system (i.e. no pasture rotation).

On all three farms the lambs were grazing with ewes from 15 to 25 April until the end of October. The farms were visited every 2 weeks and individual faecal samples were collected from the rectum of all animals on the farms on each occasion. The lambs were weighed, and their BCS and DS (i.e. faecal material adhering to the breech area) was also estimated. Three replicates of approximately 400 g samples of herbage were collected from each pasture to determine the contamination with L_3_ larvae.

### Farms and animals

On the TT farm, 24 mix-breeds of Romanov and Lithuanian black-headed sheep (10 adult and 14 lambs) grazed on the 2.3-hectare pasture (339 kg/ha). In the previous 4 years the animals had been treated with injectable ivermectin (Biomectin^®^ 1%, Poland) twice a year (spring and autumn) before turn-out and at housing according to the manufactures recommendations.

The T(S)T farm, had 57 (21 adult and 36 lambs) Lithuanian black-headed, Romanov and crossbreeds with Berrichon du Cher animals grazed on the 5.7-hectare pasture (371 kg/ha), which during the previous 8 years had been treated with injectable ivermectin as described above.

On the control (C) farm 28 animals (13 adult and 15 young animals), mostly Romanov and crossbreeds, grazed on the 3-hectare pasture (273 kg/ha). The animals had not been treated with anthelmintics the previous 6 years.

The age of lambs at the start of the study was 4–8, 2–12 and 4–8 weeks on the TT, T(S)T and C farms respectively. A total of 8 lambs both in the TT and T(S)T flocks, weighing 35–50 kg, were withdrawn from the study for slaughter from early August.

On all three farms the mating season began at the end of August or October. The animals on all farms were examined for the presence of *Fasciola* and lungworm infection on two occasions (April and September). In addition, the parasites on all farms were examined before the study for the presence of ivermectin and benzimidazole resistance using in vitro methods [11, 17]. It was then confirmed that the nematode parasites were susceptible to both anthelmintics used in this study.

The study was performed in compliance with Lithuanian animal welfare regulations (No. B1-866, 2012; No. XI-2271, 2012) and was approved by the Lithuanian Committee of Veterinary Medicine and Zootechnic Sciences (Protocol No. 07/2010).

### Treatments

Faecal samples were collected from both ewes and lambs and the average EPGs were calculated for each farm. As a part of the trichostrongylid control programme and due to *Moniezia* infection, all the adult animals were treated orally with fenbendazole at a dose rate of 7.5 mg/kg of body weight (Panacur^®^ granules, Netherlands). This was done before the grazing season (at the end of April) on both the TT and T(S)T farms and irrespective of EPG counts.

The second treatment was applied with injectable ivermectin (Biomectin^®^ 1%, Poland) subcutaneously behind the scapula at a dose of 0.2 mg/kg body weight on both farms. On the TT farm all ewes and lambs were treated on 2nd August regardless of their EPG counts, while on the T(S)T farm only those animals with an EPG ≥ 300 were treated on 1st July.

### Parasitological analyses and measurements

Faecal samples were stored at + 4 °C and analysed within 2 days using a modified McMaster technique with zinc chloride (density 1.4) and a diagnostic sensitivity of 20 EPG [29, 30].

On each sampling occasion faecal cultures were prepared in triplicates separately for ewes and lambs on each farm. Ten grams of faeces were mixed with 10 g of vermiculite and incubated for 7 days at 27 °C meanwhile water was added to maintain an adequate moisture level. Then third-stage larvae (L_3_) were recovered from the coprocultures using a Baermann technique [31]. Totally 100 L_3_ were identified to species or genera by microscopy according to [32, 33].

Three replicate herbage samples weighing approximately 400 g were collected from the pasture grazed by sheep on each farm bi-weekly. Herbage samples were collected every 10–20 steps by walking across the pasture in a W-shaped pattern. Grass within 20 cm of faecal pellets was avoided. Larvae were isolated as described in [34] and counted, and the results expressed as the number of L_3_ per kg of dried grass.

On each sampling occasion, BWG and BCS (on a scale of 1–5 with 0.5 unit intervals) were recorded for lambs, as described by Campbell et al. [35]. Dag scores were assessed according to guidelines from Australian Wool Innovation [36] on a five-point scale: 1—no dags to 5—extensive dags.

### Statistical and meteorological analyses

Descriptive statistics were calculated using Microsoft^®^ Excel 2007. The differences in FEC, weight gains in lambs, dag scores and body condition scores between farms were analysed using a repeated measures analysis of variance (ANOVA) in BMI SPSS Statistics 21 version.

Data on monthly precipitation and average temperatures were obtained from the meteorological station situated 5–15 km from the examined farms.

## Results

### Eggs in faeces

**Fig. 1:**
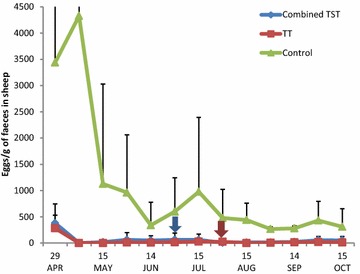
Faecal egg count in ewes. Arrows indicate the time of treatments

At the start of the study the average number of trichostrongyle EPG in ewes faeces was 283 ± 17, 378 ± 31 and 3440 ± 5865 on the TT, T(S)T and C farms, respectively. Thereafter FEC decreased in the first half of May in the control ewes, reaching its lowest level of 340 ± 437 EPG in the middle of June in comparison with TT and T(S)T farms (*P* < 0.05). Furthermore the number of trichostrongyle eggs in ewes has increased significantly (*P* < 0.01) to a level of 978 ± 1415 EPG in July on the untreated C farm compared to those on the TT and T(S)T farms. Meanwhile the FEC were very low in ewes both on the TT and T(S)T farms, and never exceeded 28 ± 31 and 63 ± 127 respectively (Fig. 1).

**Fig. 2:**
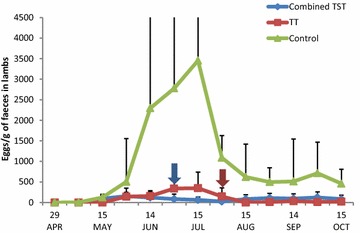
Faecal egg count in lambs. Arrows indicate the time of treatments

At the start of the study, no nematode eggs were found in the lambs on any of the farms (Fig. 2). EPG counts then increased on the C farm, reaching the peak value of 3500 ± 6950 EPG in the middle of July and was significantly higher (*P* < 0.001) when compared to those on the TT and T(S)T farms. In contrast, there were no significant differences in FEC between the TT and T(S)T farms in ewes and lambs. *Nematodirus* spp. eggs were in general consistently low and did never exceed 100 EPG on the C and TT farms. The only exception was on the T(S)T farm in June, with numbers exceeding 300 EPG in five animals.

The trichostrongyle nematode population consisted mainly of *Teladorsagia* (54.9% vs. 46.7%), *Trichostrongylus* (11.6% vs. 21.4%), *Cooperia* (15.9% vs. 20%), *Haemonchus* (8.7% vs. 6.3%), *Oesophagostomum* (6.8% vs. 4.2%) and *Chabertia* (2.1% vs. 1.4%) in ewes and lambs, respectively. *Teladorsagia* was the dominant genus on all farms. The highest prevalence of *Teladorsagia* were found in May in the ewes and lambs on the C farm (76% vs. 82%), on the TT farm (52.3% vs. 46.7%) in June and on the T(S)T farm (60.7% vs. 58%) in August. Shedding of *Haemonchus* eggs started in June and the highest prevalence was recorded on the C farm in ewes (29.4%) and lambs (30.7%) in October.

### Pasture contamination

**Fig. 3:**
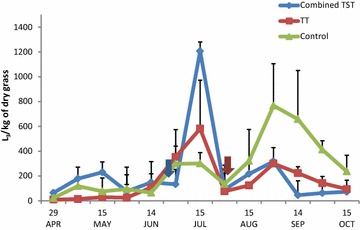
Number of L_3_ larvae on herbage. Arrows indicate the time of treatments

In early May the number of infective L_3_ stage larvae was low (118–178 L_3_/kg of dry grass) on all farms (Fig. 3). Later it peaked in the middle of July on the TT (581 L_3_/kg of dry grass) and T(S)T (1206 L_3_/kg of dry grass) farms, while pasture contamination on the C farm consistently increased during the grazing season to a peak value of 767 L_3_/kg of dry grass in early September. Infective larvae on pastures of the TT, T(S)T and C farms consisted of *Teladorsagia* (42.3, 48.7 and 66.2%), *Nematodirus* (41.7, 28.8 and 13.7%), *Trichostrongylus* (6.4, 6.1 and 5.0%), *Cooperia* (7.9, 7.3 and 2.7%), *Haemonchus* (0, 0 and 12.1%), *Oesophagostomum* (1.7, 6.3 and 0.3%) and *Chabertia* (0, 2.8 and 0%) respectively.

### Meteorological and other observations

During the study rainfall was elevated, with precipitation of around 83, 84 and 116 mm when compared with an average value of 54, 63 and 74 mm in May, June and August respectively. The temperature was slightly elevated (20.9 and 18.5 °C) on all farms when compared with an average value of 17.1 and 16.2 °C in July and August respectively. There was a constant snow cover on the pastures from the end of December until the end of March before the study.

### Mean weight gains in lambs

**Fig. 4:**
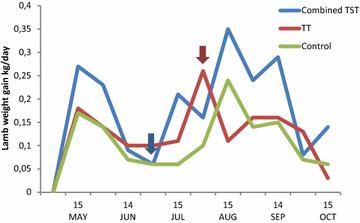
Lamb weight gain kg/day. Arrows indicate the time of treatments

The average mean daily BWG were comparable in lambs on the control farm (0.17 kg/day) and the TT farm (0.18 kg/day) during the first 2 weeks of May, whereas it was significantly (*P* < 0.001) higher (0.27 kg/day) on the T(S)T farm. In July BWG decreased to 0.1, 0.06 and 0.06 kg per day in the TT, T(S)T and control farms respectively. From then on the BWG recovered in all groups to a level of 0.26, 0.35 and 0.24 kg per day in the TT, T(S)T and C farms respectively in the first half of August, followed by a decrease towards the end of the grazing season (Fig. 4).

Following the second treatment, the average BWG increased from 0.16 to 0.24 kg/day on the T(S)T farm (*P* < 0.05), while they remained comparable (0.15 kg/day vs. 0.14 kg/day) in the animals on the TT farm. Meanwhile the average BWG has decreased (0.13 kg/day vs. 0.12 kg/day) on the C farm. Accordingly, the average BWG were significantly higher both on the T(S)T (*P* < 0.001) and TT farms (*P* < 0.01) than on the C farm.

### Body condition score

Average BCS in lambs were 3.1 ± 0.28, 3.59 ± 0.32 and 2.89 ± 0.23 on the TT, T(S)T and C farms respectively. Following the second treatment on the T(S)T farm, the BCS has improved gradually (*P* > 0.05) from 3.4 to 3.8 ± 0.12 in lambs, while after some fluctuations BCS remained at 3.1 in the lambs on the TT farm. The average BCS in ewes followed a similar pattern to those of lambs. Following the second treatment the BC score improved gradually from 3.1 to 3.3 ± 0.25 in ewes on the TT farm, while BCS remained at a comparable level of 3.9 ± 0.55 on the T(S)T farm. The average BCS was significantly (*P* < 0.05) higher on the T(S)T and TT farm when compared to those on the C farm.

### Dag scores

At the start of the study the average DS on the C farm were high both in lambs (3.2 ± 0.5) and ewes (3.4 ± 0.5), while animals on the TT and T(S)T farms were in general cleaner. Both on the TT and T(S)T farms DS remained negligible throughout the study and never exceeded 2 (lightly dagged). The only exception was on the T(S)T farm where some lambs had the highest dag score (3) starting from August until the end of grazing season. However, the average dag score in lambs on the T(S)T farm remained below 2. On the C farm at least one animal had a dag score of 3 or 4 (extensively dagged), including lambs and adult animals at every estimation point during the grazing season.

## Discussion

Traditionally a strategic treatment approach with up to two to three treatments per year in ewes is used on most Lithuanian sheep farms [18, 22]. The main purpose of targeted (selective) treatment approaches is to optimise anthelmintic usage, leave some parasites *in refugia* and thereby slow down the selection for anthelmintic resistance [19]. Maintenance of the parasite population *in refugia* is now considered one of the most important factors in slowing down the development of AR and should be included in any potential prophylactic control programme suggested for nematode parasites [21]. In the present study, combinations of strategic and tactical treatments were evaluated in comparison with untreated control animals.

The larvae of *Teladorsagia* survive on pasture during the winters in Lithuania. This results in an early pasture contamination with infective L_3_, as has previously been recorded in Lithuania on pastures grazed by sika deer [37] and goats [38]. The initial aim of the present study was to avoid early pasture contamination by ewes at the start of the grazing season. To achieve this, all ewes on the TT and T(S)T farms were treated with fenbendazole before turn-out.

In the present study*, Teladorsagia* was the most prevalent genus, irrespective of the farm and age of animals. The highest concentration of *Teladorsagia* L_3_ larvae were isolated from grass sampled in May. Infective larvae of *Haemonchus* were only observed on the control farm (16.3% in lambs and 22.4% in ewes faeces, respectively), resulting in consistently low (12.1%) pasture contamination during the study.

At the start of the study, we observed a dramatic increase in FEC of the ewes. This rise was very pronounced especially on the C farm (3440 ± 5865 EPG), while it was significantly lower (*P* < 0.001) on the TT (283 ± 17 EPG) and T(S)T (378 ± 31 EPG) farms. After treatment of ewes in the spring FEC decreased and never exceeded 28 ± 31 and 63 ± 127 on the TT and T(S)T farms respectively. As anticipated before the study, the spring treatment resulted in lower pasture contamination with L_3_ both on the TT and T(S)T farms than on the C farm, thus preventing larval exposure resulting in high infection levels in lambs. However, grass height was significantly reduced due to low precipitation and elevated temperatures on the T(S)T and TT farms in July, resulting in a significant increase in pasture contamination for a short period in the middle of July. This could also partly be attributed to higher grazing pressure on both farms when compared to those on the control farm.

The clinical manifestation of trichostrongylid infection can usually be observed in young kids, since a fully expressed immune response against GIN normally appears at the age of 12 months [1]. The EPG of trichostrongylids was also significantly higher in lambs compared to ewes. The number of trichostrongylid eggs in lambs was highest on the control farm during the study when compared to those on the TT and T(S)T farms (*P* < 0.001) due to continuous pasture contamination. On the C farm FEC increased steeply (*P* < 0.001) until the end of July, reaching a peak of 3500 ± 6950 EPG. However, this was attributed to a few lambs on the C farm having extremely high EPG counts (> 10,000). The dominant GIN genera on the control farm in lambs during July were *Teladorsagia* but to a lesser extend also *Haemonchus*. However, due to low infection level the clinical signs (anaemia) related to *Haemonchus* infection in lambs was not recorded on C farm.

On the TT farm, all animals were treated a second time on 2nd August irrespective of EPG counts. In contrast, the need for a second treatment was assessed based on FEC on the T(S)T farm and therefore only animals with ≥ 300 EPG were treated. Following the second treatment in August, the EPG on the TT farm decreased and remained below 33 for the remainder of the study. On the T(S)T farm, 14.3% of ewes and 25.0% of lambs with EPG ≥ 300 were treated in July. However, the average FEC on the TT (32.7 and 93.5) and T(S)T farms (60.5 and 79.2) were comparable at every sampling occasion, while it was significantly higher (*P* < 0.05) both in the ewes (845) and lambs (1109) on the C farm.

Despite differences in the quality of pasture and grazing density, the average BWG at housing were highest in lambs on the T(S)T farm (*P* < 0.001), followed by those on the TT (*P* < 0.001) farm, when compared to those on the C farm. Due to the low grazing pressure, the pasture contamination with L_3_ was low but also delayed on the C farm. However the control animals still developed clinical signs of GIN infection. The spring treatment of the ewes on the TT and T(S)T farms clearly had a significant impact and reduced the shedding of nematode eggs on both farms. Thus, this had a beneficial impact and lowered the pasture contamination with infective L_3_ at the start of the grazing season, while it remained significantly higher on the C farm. The positive effects of the spring treatment of ewes were also reflected in increased BWG in lambs during the first half of the grazing season. Following the second treatment BWG in lambs on the T(S)T farm peaked (from 0.16 to 0.24 kg) (*P* < 0.05) when compared to lambs on the TT farm, while the control lambs gained weight but at a slower rate. These differences were also substantiated by the BC scores of lambs, which were highest on the T(S)T farm when compared to those on the control and TT farms (*P* < 0.05).

## Conclusions

The results of this study show that targeted (selective) treatment strategy is useful in reducing the clinical effects of gastrointestinal parasitism and increased the performance especially of the lambs. On the T(S)T farm some animals were left *in refugia*. T(S)T also resulted in 77% less anthelmintics being used compared to the TT farm, which in itself may slow down the selection for AR. However, more precise studies are required involving larger flocks on comparable pastures before any final recommendations for the application of T(S)T strategies can be made.

## Abbreviations

AR: anthelmintic resistance
BCS: body condition score
BWG: body weight gain
DS: dag score
EPG: eggs/g faeces
FEC: faecal egg count
GIN: gastrointestinal nematodes
T(S)T: targeted selective treatment
TT: targeted treatment
WGS: world geodetic system
